# Remote estimation of leaf area index (LAI) with unmanned aerial vehicle (UAV) imaging for different rice cultivars throughout the entire growing season

**DOI:** 10.1186/s13007-021-00789-4

**Published:** 2021-08-10

**Authors:** Yan Gong, Kaili Yang, Zhiheng Lin, Shenghui Fang, Xianting Wu, Renshan Zhu, Yi Peng

**Affiliations:** 1 School of Remote Sensing and Information Engineering, Wuhan University, Wuhan, China; 2 College of Life Sciences, Wuhan University, Wuhan, China; 3 Lab for Remote Sensing of Crop Phenotyping, Wuhan University, Wuhan, China

**Keywords:** Leaf area index, Rice phenology, Unmanned aerial vehicle, Vegetation index, Canopy reflectance, Canopy height

## Abstract

**Background:**

Rice is one of the most important grain crops worldwide. The accurate and dynamic monitoring of Leaf Area Index (LAI) provides important information to evaluate rice growth and production.

**Methods:**

This study explores a simple method to remotely estimate LAI with Unmanned Aerial Vehicle (UAV) imaging for a variety of rice cultivars throughout the entire growing season. Forty eight different rice cultivars were planted in the study site and field campaigns were conducted once a week. For each campaign, several widely used vegetation indices (VI) were calculated from canopy reflectance obtained by 12-band UAV images, canopy height was derived from UAV RGB images and LAI was destructively measured by plant sampling.

**Results:**

The results showed the correlation of VI and LAI in rice throughout the entire growing season was weak, and for all tested indices there existed significant hysteresis of VI vs. LAI relationship between rice pre-heading and post-heading stages. The model based on the product of VI and canopy height could reduce such hysteresis and estimate rice LAI of the whole season with estimation errors under 24%, not requiring algorithm re-parameterization for different phenology stages.

**Conclusions:**

The progressing phenology can affect VI vs. LAI relationship in crops, especially for rice having quite different canopy spectra and structure after its panicle exsertion. Thus the models solely using VI to estimate rice LAI are phenology-specific and have high uncertainties for post-heading stages. The model developed in this study combines both remotely sensed canopy height and VI information, considerably improving rice LAI estimation at both pre- and post-heading stages. This method can be easily and efficiently implemented in UAV platforms for various rice cultivars during the entire growing season with no rice phenology and cultivar pre-knowledge, which has great potential for assisting rice breeding and field management studies at a large scale.

## Background

With the ever-increasing global population, the food demand continues rising all over the world. Moreover, the declining in arable land, conjugated with more frequent extreme climate events and severe environmental pollutions, poses tough challenges to feed the growing world and ensure food security. Rice is one of the most important grain crops providing food for more than half of the world’s people [1]. A great number of studies have been dedicated to the improvement of rice production and quality [2–4].

Leaf area index (LAI) is defined as the total one-sided area of leaf tissues per unit ground surface area [5–7], which closely relates to canopy-environment exchange processes such as light absorption, water interception, evapotranspiration and carbon uptake [6–9]. As a good indicator of canopy photosynthesis capacity, LAI during the growing season strongly influences crop production [10, 11]. The dynamic monitoring of LAI can provide valuable information to investigate crop growth in the response of ambient environment thus evaluating its final yield [12–14].

The direct methods usually obtain vegetation LAI by means of manually sampling leaves and measuring their total area using leaf area meter, which is destructive, time-consuming and labor-intensive [6, 15]. Some devices were invented based on indirect optical methods to infer LAI from measured radiation transmission through the canopy [15–17], offering good alternatives to traditional destructive methods. But such devices have to be operated manually underneath the canopy in a stop-and-go mode, which still require extensive field work and not suitable for agricultural applications at a large scale [18–20].

Recently, the use of remote sensing technology to estimate vegetation LAI has been widely developed especially for large-scale and long-term crop monitoring [21, 22]. Canopy reflectance, which can be recorded by remote sensors at various platforms from close range to satellite altitude, is mostly governed by vegetation absorption and scattering that highly correlated with crop LAI [13, 23]. Vegetation indices (VI) formulated from math combinations of reflectance at several bands [24, 25], as well as many multiple regression and machine-learning methods using multi- or hyper-spectral reflectance [26], were successfully developed to extract the most useful spectral information for LAI estimation. Nguy-Robertson et al. [27] developed combined VI based on visible and NIR reflectance measured by ground-mounted radiometers to estimate LAI in maize and soybean; Yao et al. [28] estimated wheat LAI using modified triangular VI obtained from unmanned aerial vehicle (UAV) multispectral imaging; Kira et al. [29] applied support vector machines (SVM), neural network (NN), multiple linear regression (MLR) and VI techniques to estimate soybean and maize green LAI with satellite reflectance products; Wang et al. [30] compared MLR, partial least squares (PLS) regression and least squares support vector machines (LS-SVM) methods to determine rice LAI using reflectance of selected optimal wavebands from hyperspectral spectroradiometer. In addition to spectral information, canopy structure information was also useful to indicate vegetation biophysical parameters [31]. It is found that plant height showed a significant relationship with biomass in winter wheat [19]. The 3D point clouds data generated from UAV-based multispectral imagery, accounting for canopy thickness, height and leaf density distribution, was employed to estimate grape LAI [31]. Remote sensing gives a fast, non-destructive and relatively cheap solution for monitoring crop LAI that can be extended to a regional scale [7, 13].

The remote estimation of vegetation LAI has been studied for decades, but the routine and generic model that can achieve high accuracy for various species and field conditions is yet to be developed [15, 21, 32]. It is found that the phenological stage of the vegetation is one factor influencing the performance of vegetation parameter estimations using canopy reflectance [22], especially for crop having prominent flowers, fruits or grains during its growing season [27]. The VI-based methods have a long history for its high efficiency and simplicity used in a wide variety of terrestrial science applications to characterize the Earth’s vegetation cover from space [33–35]. But the progressing phenology affects relationships between VIs and biophysical parameters throughout the vegetation growing season particularly for crops with their distinct phenology-related features [22, 36]. It is observed that there were obvious differences in the VI vs. canopy chlorophyll content relationships between the vegetative and reproductive stages in maize and soybean [37]. Fang et al. [38] found the uncertainties increased by 50% when using VI for estimating vegetation fraction in oilseed rape during its flowering season. Many studies showed that in rice VIs worked well for biomass estimation only for the pre-heading stages, but they were weakly related to biomass at post-heading stages [14, 39]. Zheng et al. [40] and Zha [41] reported the correlation between nitrogen concentration and VI was low after panicles emerging out from the sheath.

Using remote sensing for LAI estimation has been successfully employed in many vegetation types [32, 36], but VI-based methods in rice are mostly applied for a few cultivars or for specific growth period. Rice has unique canopy features during reproductive and ripening stages. With the panicle exsertion, green and erect panicles occupy most of the top canopy. As grain growing toward maturity, the panicles become droopy with increased weight and their color turn to yellow [42, 43]. The changes in the structure and color of panicles make the canopy reflectance complicated after rice heading. Since VIs are calculated from canopy reflectance, VI-based models and algorithms for rice LAI estimation are greatly influenced by phenology factor and may be specific to different growth stage thus limiting their application throughout the entire growing season. However, it involves field work to visually determine rice phenology stage which is sometimes slow and subjective [44, 45]. And with the advancement of breeding technology, hundreds of rice cultivars have been bred with very different phenology cycles [44–46]. Thus, it is unrealistic to firstly get the pre-knowledge of rice phenology and then calibrate the model for each phenology stage. The model that are generic and able to efficiently monitor rice LAI of different cultivars and for the entire growing season is pressing especially for the study site having a number of different rice cultivars such as large breeding nursery.

UAV imaging is increasingly used as an emerging technology applied in the field of precision agriculture due to its high resolution, reduced cost and better flexibility of flight and sensor settings [47, 48]. For example, López-Granados et al. [49] detected weed in croplands based on UAV images acquired at different heights; Yang et al. [11] predicted rice grain yield at the ripening stage with UAV RGB images; Hu et al. [50] recognized the diseased *Pinus* trees in UAV images to help monitor and control tree diseases in large areas; Meinen et al. [51] mapped erosion and deposition in an agricultural landscape based on UAV images obtained by four acquisition schemes. UAV-collected data is becoming an effective and convenient tool to evaluate crop growth for assisting in farm managements and crop breeding studies [52, 53].

Canopy spectra reflects crop absorption-related biological properties, and plant structure may indicate phenology-related morphological traits. The structure information retrieved from UAV imaging may be a good addition to canopy reflectance for accurate LAI estimation in rice of a variety of cultivars at different phenology stages. In this study, we aim to explore a simple method to remotely estimate LAI with UAV imaging for multiple rice cultivars throughout the entire growing season. Our objectives are to: (1) evaluate several widely used VI for LAI estimation at different phenology stages, (2) analyze how phenology factor affected remotely sensed canopy spectral and structure signal, and (3) develop and improve VI-based model for estimating rice LAI, not requiring algorithm re-parameterizations for different phenology stages.

## Materials and methods

### Study area

**Fig. 1:**
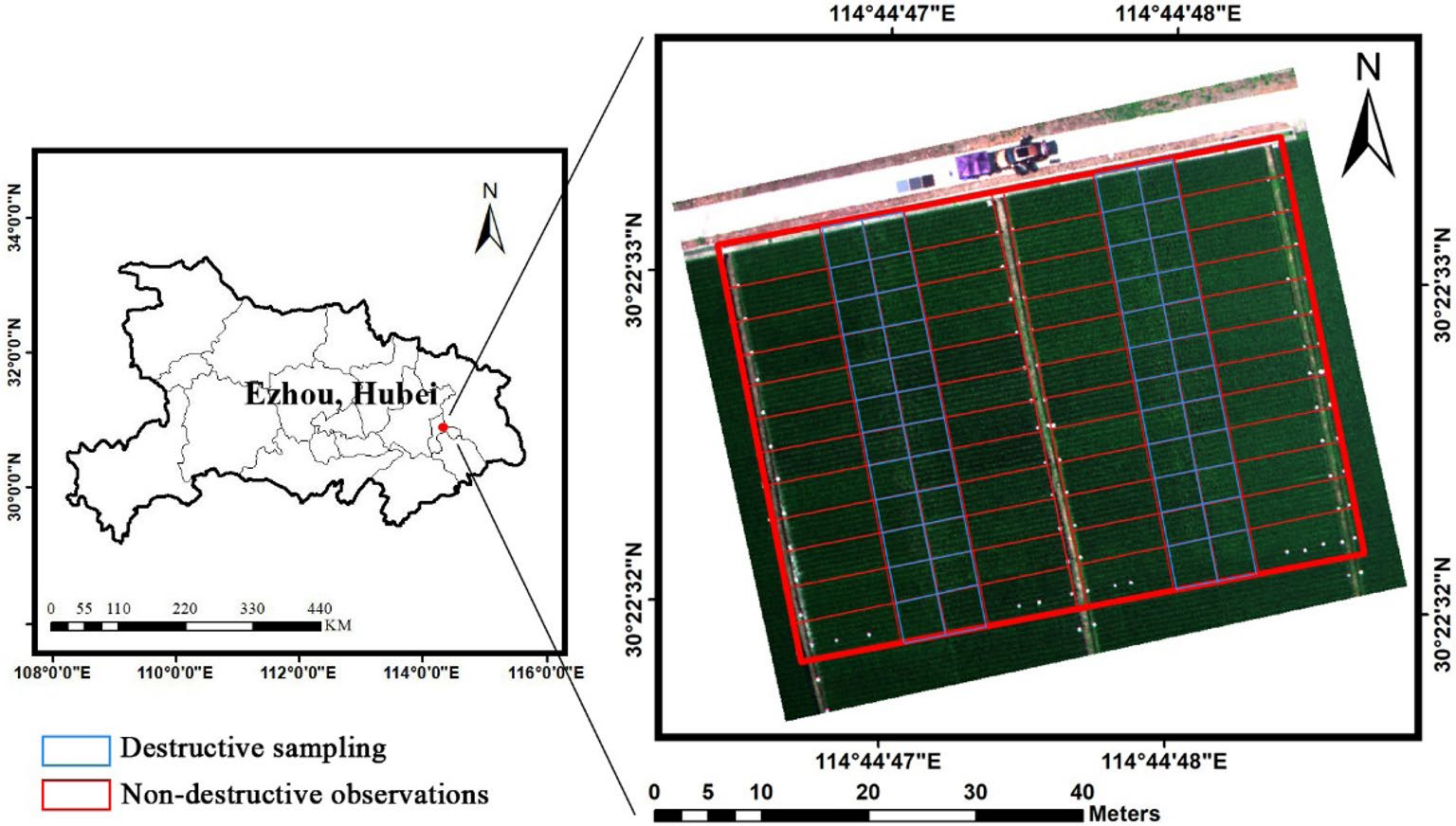
Study Area of 48 rice cultivars in Ezhou, Hubei, China

The study site was located at the Hybrid Rice Experiment and Research Base of Wuhan University near Ezhou City, Hubei Province, China (30°22′33″ N, 114°44′48″ E) (Fig. 1). The flat terrain, subtropical monsoon climate of this area is suitable for rice growth. The annual average temperature, sunshine time, and precipitation are 17 °C, 2003.8 h, and 1282.8 mm, respectively. This study site has 48 plots planted with different hybrid rice cultivars, which applied the same planting density, nitrogen fertilizer and the standard local field managements. The rice varieties belong to the Honglian type hybrid rice, which are widely planted in the Middle-lower Yangtze Plain in China due to their high yield, wide adaptability and good temperature and disease resistance [54]. These 48 cultivars were selected in our study site since they are representative in China and have quite obvious morpho-physiological differences. The maximum LAI of these cultivars ranged from 2.76 to 8.53, and their height ranged from 0.82 to 1.13 m after the maturity. They were sown on May 11th, 2019, and the seedlings of 48 rice cultivars were transplanted to the experiment field on June 9th, 2019 with one cultivar in each plot. For each plot, six rows were planted and each row had double lines. The distance between the rows was 33 cm and 20 cm within the row. Before transplanting, several whiteboards were erected on the edge of the plots to help locate different plots in the images. After transplanting, each plot was divided into a subplot (4 m × 3 m) for destructive sampling (around 270 bundles) and a subplot (8 m × 3 m) for non-destructive observations (Fig. 1), trying to reduce the impact of destructive sampling on remotely sensed canopy spectra. Thirteen field campaigns for LAI measurements and UAV images of the study site were carried out throughout the entire rice growing season from June to September (Date: June 26th, July 2nd, July 6th, July 14th, July 22nd, July 27th, Aug 1st, Aug 6th, Aug 11th, Aug 16th, Aug 22nd, Aug 29th, and Sep 3rd). For each campaign, the UAV flight was firstly arranged to acquire site images and plant sampling was then conducted in the field.

### Destructive measurements of LAI

For each campaign, three bundles of rice plants in the non-edge area of each plot were sampled for destructive measurements of LAI. The plants were placed in the cooler with ice bags and transported to the laboratory for further destructive vegetation measurements. The green leaves were cut from the plant and run through LI-3100C leaf area meter (LI-COR, Lincoln, NE, United States). The leaf area (LA) of each plot was obtained by the sum of all leaf areas of three bundles, and then plot LAI was calculated as: $LAI = \frac{LA}{n} \times \rho$*,* where $n$ is the number of samples in each plot and $\rho$ is the plant density per square meter. In this study, n was equal to 3 and $\rho$ was equal to 22.5 bundles/m^2^.

### Manual determination of heading date in rice

Heading date in rice is generally defined as the time when approximately 50% of the panicles have exserted [43, 45], which was determined by manual visual observations in the field. The heading date of the studied 48 rice cultivars varied between 59 and 73 Days After Transplanting (DAT). In this study, the growing season of each rice cultivar can be roughly divided based on heading date into pre-heading stages and post-heading stages.

### Canopy reflectance, vegetation index and canopy height remotely derived from UAV images

For each campaign, two UAV flights were arranged to acquire canopy spectral and structure information respectively. On the first flight the 12-band multispectral images were obtained for the study site and the RGB images was then taken on the other flight. The parameters of sensors used in two UAV flights were summarized in Table 1.

**Table 1:** Two UAV flights with different sensors

	RGB images	12-Band images
Types of cameras	DJI FC 6310 camera	Mini-MCA camera system
Central wavelength	R, G, B	490 nm, 520 nm, 550 nm, 570 nm, 670 nm, 680 nm, 700 nm, 720 nm, 800 nm, 850 nm, 900 nm, 950 nm
Image size	5472 × 3648	1280 × 1024
Image resolution	0.8 cm/pixel	5.5 cm/pixel
Fight height	30 m	100 m
Field of view	84°	Horizontal angle of view: 38.26° Vertical angle of view: 30.97°
Information retrieved from images	Crop surface model	Multi-spectral canopy reflectance

**Fig. 2:**
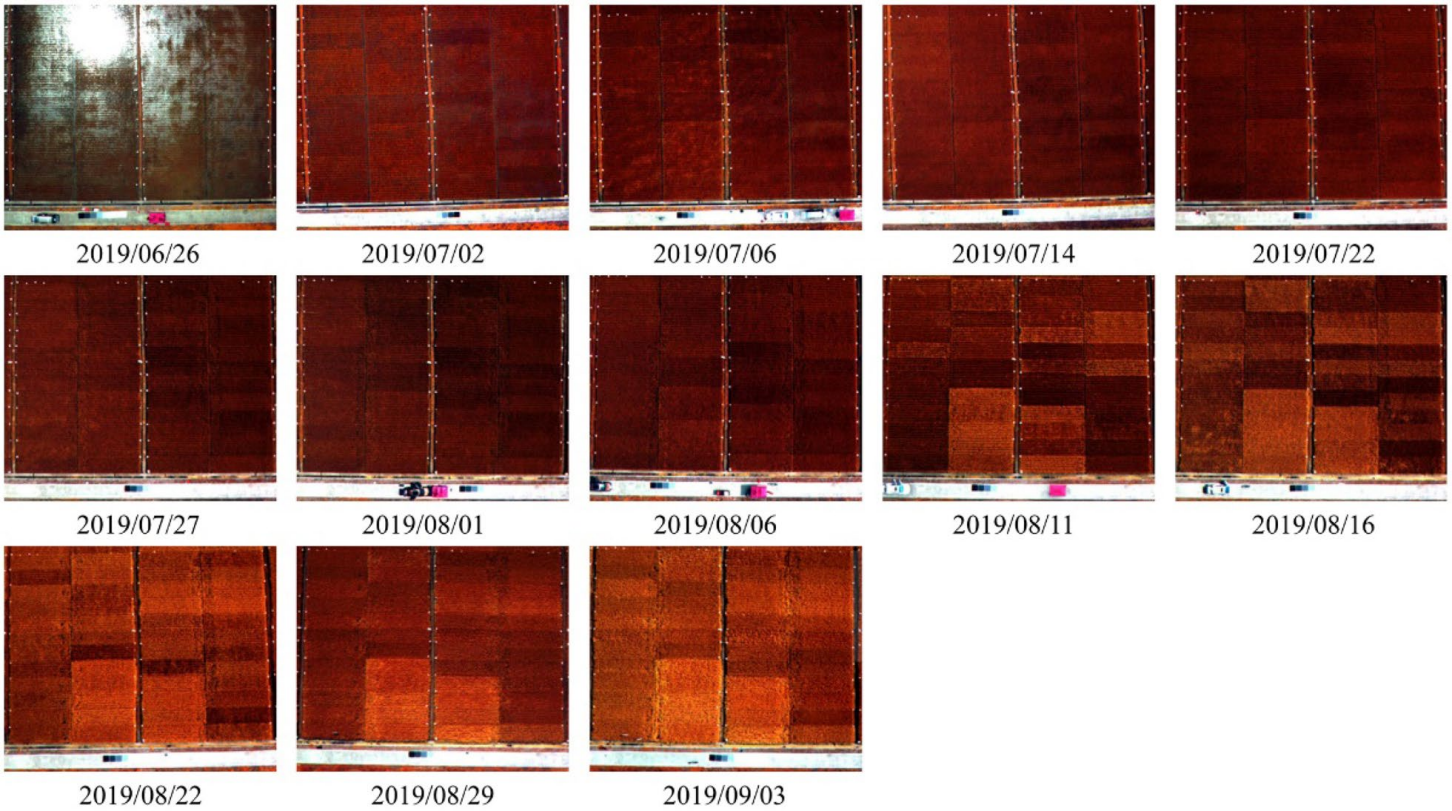
The multi-spectral images taken throughout the growing season for the study site (The standard false color composite images are shown)

The 12-band images of the study site were obtained by a Mini-MCA camera system (Tetracam Inc., Chatsworth, CA, United States) mounted on M8 UAV (Beijing TT Aviation Technology Co., Ltd.). This 12-lens camera system was equipped with customer-specified band pass filters centered at a wavelength of 490 nm, 520 nm, 550 nm, 570 nm, 670 nm, 680 nm, 700 nm, 720 nm, 800 nm, 850 nm, 900 nm, and 950 nm, respectively. The 12 camera lens were co-registered in the laboratory prior to the flight so that corresponding pixels of each lens were spatially overlapping in the same focal plane [34]. The flights were taken in the sunny and cloudless weather between 10:00 AM and 2:00 PM, with the altitude of 100 m and the image spatial resolution of 5.5 cm. A gimbal stable platform was installed on UAV to adjust the camera system pointing close to nadir during the flight. For image radiometric calibration, a linear relationship was assumed between surface reflectance ($\rho$) and image digital numbers (DN) as [55, 56]:

1 $$\rho_{\lambda } = DN_{\lambda } \times Gain_{\lambda } + Offset_{\lambda }$$

where $\rho_{\lambda }$ and $DN_{\lambda }$ were the surface reflectance and corresponding image digital numbers at wavelength $\lambda$. Eight near-Lambertian calibration canvases, at the constant reflectance of 0.03, 0.06, 0.12, 0.24, 0.36, 0.48, 0.56 and 0.80, were placed in the camera’s field of view to solve $Gain_{\lambda }$ and $Offset_{\lambda }$ value in different bands using the least-square method [57, 58] for image radiometric calibration. In this case, the canopy reflectance at 12 bands can be calculated based on Eq. (1). The images of canopy reflectance taken throughout the growing season were shown in Fig. 2.

The RGB images of the study site with DJI FC 6310 camera mounted on DJI Phantom 4 Professional UAV (SZ DJI Technology Co., Ltd., Shenzhen, China) for canopy 3-D reconstruction. The field of view of the camera was 84°, and the image resolution was 0.8 cm. Using Agisoft Photoscan Professional v1.4.5 (Agisoft LLC, St. Petersburg, Russia), the canopy Digital Surface Model (DSM) was generated [59–62]. The canopy height (H) was calculated as:

2 $$H = DSM - DSM_{soil}$$

where $DSM_{soil}$ is equal to the DSM on the date before rice transplanting. This approach is widely applied to retrieve canopy height with the accuracy around 3 cm [62–64].

The 12-band images taken on the first flight (Jun. 26) was greatly affected by water high specular reflectance (bright spots in Fig. 2) since the rice seedlings were just transplanted in the study site and the water was not drained away completely. The pixel having DN values greater than 250 in all bands was marked as “bad pixel” and excluded for further calculation. The plot with observation area including more than 100 such bad pixels was not considered as valid sample. Thus for this image, only 24 plots were retained for model development. As water dried up in the study site, the images of other 12 flights were little affected by water and all plots could be used as valid samples.

For each rice plot, a rectangular region of interest (ROI) of the same size was defined that maximally fit the plot. The ROI included three rows of rice corresponding around 800 pixels in the 12-band image and 2000 pixels in the RGB image. The average reflectance and the average height of all pixels within the ROI were taken as the plot-level canopy reflectance and canopy height, respectively. The plot level vegetation indices (VI) were calculated from plot-level canopy reflectance. Eight VIs, which are widely used for LAI estimation in many studies [14, 18, 22, 24, 36, 57, 65, 66] and can be easily applied in current satellite sensors, were tested in this study for analysis (Table 2).

**Table 2:** Vegetation Indices (VI) tested in this study

VI	Formula	References
NDVI	$(\rho_{NIR} - \rho_{red} )/(\rho_{NIR} + \rho_{red} )$	Rouse et al. [71]
EVI2	$2.5 \times (\rho_{NIR} - \rho_{red} )/(1 + \rho_{NIR} + 2.4 \times \rho_{red} )$	Jiang et al. [35]
WDRVI	$(\alpha \times \rho_{NIR} - \rho_{red} )/(\alpha \times \rho_{NIR} + \rho_{red} )$, $\alpha = 0.2$	Gitelson et al. [72]
${\text{CI}}_{{{\text{green}}}}$	$\rho_{NIR} /\rho_{green} - 1$	Gitelson et al. [73]
${\text{CI}}_{{{\text{rededge}}}}$	$\rho_{NIR} /\rho_{red edge} - 1$	Gitelson et al. [73]
NDRE	$(\rho_{NIR} - \rho_{red edge} )/(\rho_{NIR} + \rho_{red edge} )$	Gitelson et al. [74]
MTCI	$(\rho_{NIR} - \rho_{red edge} )/(\rho_{red edge} - \rho_{red} )$	Dash et al. [75]
OSAVI	$(1 + 0.16) \times (\rho_{NIR} - \rho_{red edge} )/(\rho_{NIR} + \rho_{red edge} + 0.16)$	Steven et al. [76]

Noted that the Mini-MCA sensor sensitivity drops around 450 nm and the signal to noise ratio is relatively low at blue band [67]. On the other hand, rice reflectance at blue band is quite low due to vegetation high absorption of visible radiation [68, 69], thus at blue band the quality of Mini-MCA images was not good. So in this study we avoided the VIs with blue reflectance. EVI2 was used instead of EVI, since these two indices appeared very close in many studies [35, 68–70].

### Algorithm development for LAI estimation

In this study, the k-fold cross validation procedure [77] was used to develop the algorithms for LAI estimation. The samples were randomly split into k mutually exclusive sets (k = 10 in this study, which is a common number used in many studies [29, 65, 77, 78]) and they were trained and validated for k times. For each time, k-1 sets are used iteratively as training data for calibrating the coefficients (Coef_i_) of the relationship, and the remaining set is used as the validation data to obtain estimation accuracy: Root mean square error (RMSE_i_), coefficient of variation (CV_i_) and Bias_i_ [27, 28]. This procedure was repeated k times, with each of the k sets used exactly once as the validation data. The results form k iterations then can be averaged to produce a single estimation [65]:

3 $$Coef = \frac{1}{k}\mathop \sum \limits_{i = 1}^{k} Coef_{i} ; RMSE = \frac{1}{k}\mathop \sum \limits_{i = 1}^{k} RMSE_{i} ; CV = \frac{1}{k}\mathop \sum \limits_{i = 1}^{k} CV_{i} ; Bias = \frac{1}{k}\mathop \sum \limits_{i = 1}^{k} Bias_{i}$$

where $$CV_{i} = \frac{{RMSE_{i} }}{{Mean\left( {Measured\,LAI} \right)}}$$ and $$Bias_{i} = \frac{{\mathop \sum \nolimits_{j = 1}^{n} \left( {Estimated\,LAI_{j} - Measured\,LAI_{j} } \right)}}{n}$$

## Results

### Relationships of VI vs. rice LAI throughout the entire growing

Eight VIs were related to LAI in this study, and it is found that the correlations between rice LAI and these VIs throughout the entire growing season were quite low not exceeding 0.4 (Table 3). The ratio indices (MTCI, CI_green_ and CI_red edge_) had the lowest correlation with LAI (R^2^ around 0.14), and R^2^ of the normalized indices (NDRE, WDRVI and NDVI) appeared a little higher (R^2^ around 0.16), while EVI2 and OSAVI had relatively highest R^2^ (R^2^ = 0.2 for OSAVI and 0.38 for EVI2).

**Table 3:** Correlation (R^2^) between LAI and VI in rice of the entire growing season

	MTCI	CI_green_	CI_red edge_	NDRE	WDRVI	NDVI	OSAVI	EVI2
R^2^	0.13	0.14	0.15	0.15	0.16	0.17	0.20	0.38

**Fig. 3:**
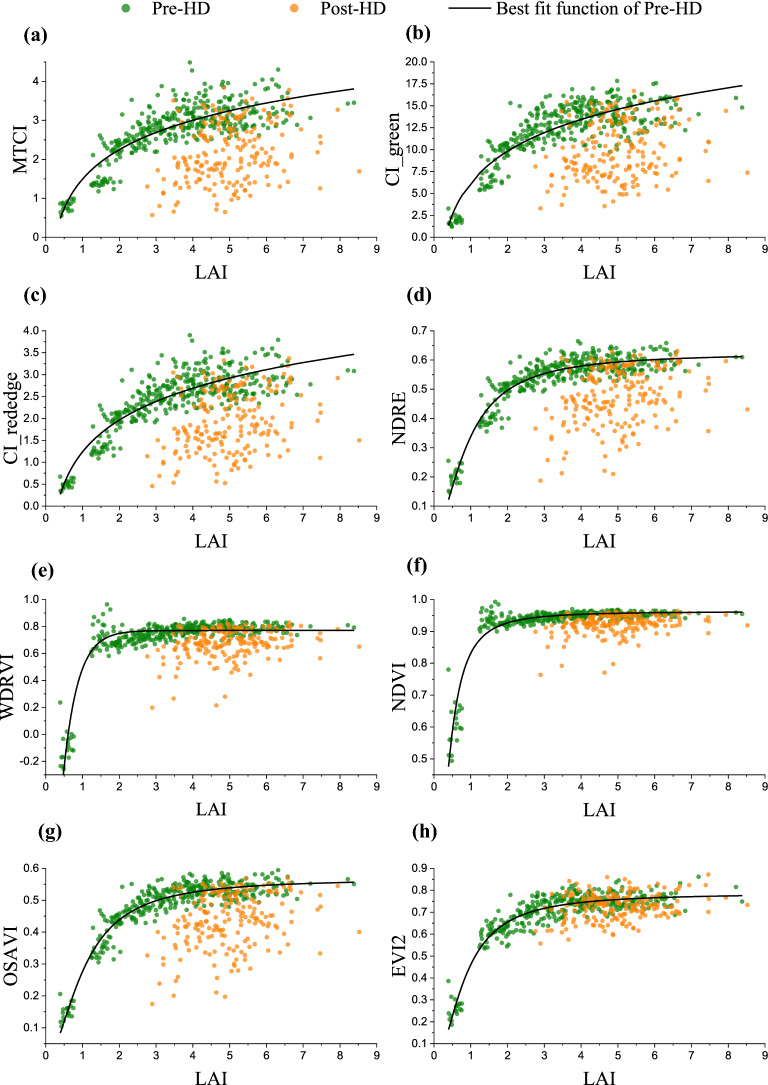
The variation of LAI plotted with **a** MTCI, **b** CI_green_, **c** CI_red edge_, **d** NDRE, **e** WDRVI, **f** NDVI, **g** OSAVI and **h** EVI2 in rice during the entire growing season. For all tested VIs, samples of post-heading (Post-HD) stages were deviated from the LAI vs. VI relationship of pre-heading (Pre-HD) stages

Figure 3 presented the variation of VI plotted with rice LAI of the entire season. It is observed that for all our tested indices the samples obviously followed two different patterns, one with samples collected before rice heading and the other with samples after heading. Thus, using one relationship for the entire rice-growing season could not well describe the LAI variation by VI (Table 3). When separating samples by heading date, during the pre-heading stages (green points in Fig. 3) all tested VIs were closely related with LAI. This agreed with the observations in many previous studies that there existed significant relationships between those VIs and LAI. But for post-heading rice samples (yellow points in Fig. 3), the relationship VI vs. LAI had high uncertainties. Also noted that before the rice heading stage, NDRE, WDRVI, NDVI, OSAVI, and EVI2 showed obvious saturation to moderate-to-high LAI variation (Fig. 3d–h), while MTCI, CI_green_, and CI_red edge_ more linearly related to LAI (Fig. 3a–c).

**Fig. 4:**
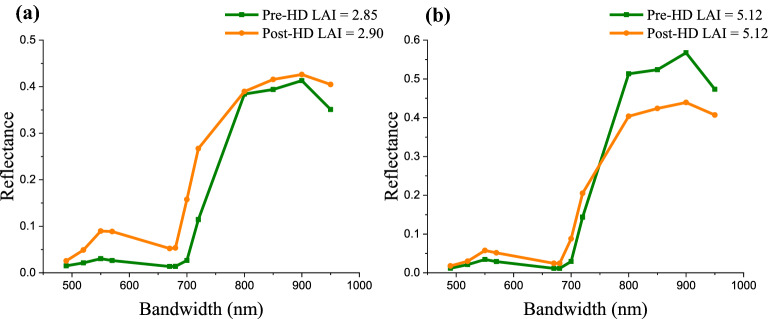
The canopy spectra of two samples with similar LAI values, **a** LAI around 2.9 and **b** LAI around 5, in pre-heading (Pre-HD) and post-heading (Post-HD) stages

The canopy spectra, of two samples with similar LAI values but one collected before rice heading and the other after rice heading, were compared (Fig. 4). For low to moderate LAI (LAI up to 3) with similar LAI, reflectance in bands of 450–800 nm after rice heading was much higher than before heading, and reflectance in bands of 800–950 nm was close between two stages (Fig. 4a). It is observed that the green and red reflectance after heading can be more than twice as that before heading. For moderate to high LAI (LAI above 5) with similar LAI, reflectance in bands of 450–750 nm was higher after rice heading while the reflectance in bands of 750–950 nm was lower. The difference in reflectance with similar LAI but at different phenology stages (Fig. 4) caused substantial hysteresis of VI vs. LAI relationships between pre-heading and post-heading stages (Fig. 3).

### LAI, VI and canopy height variation throughout the rice growing season

**Fig. 5:**
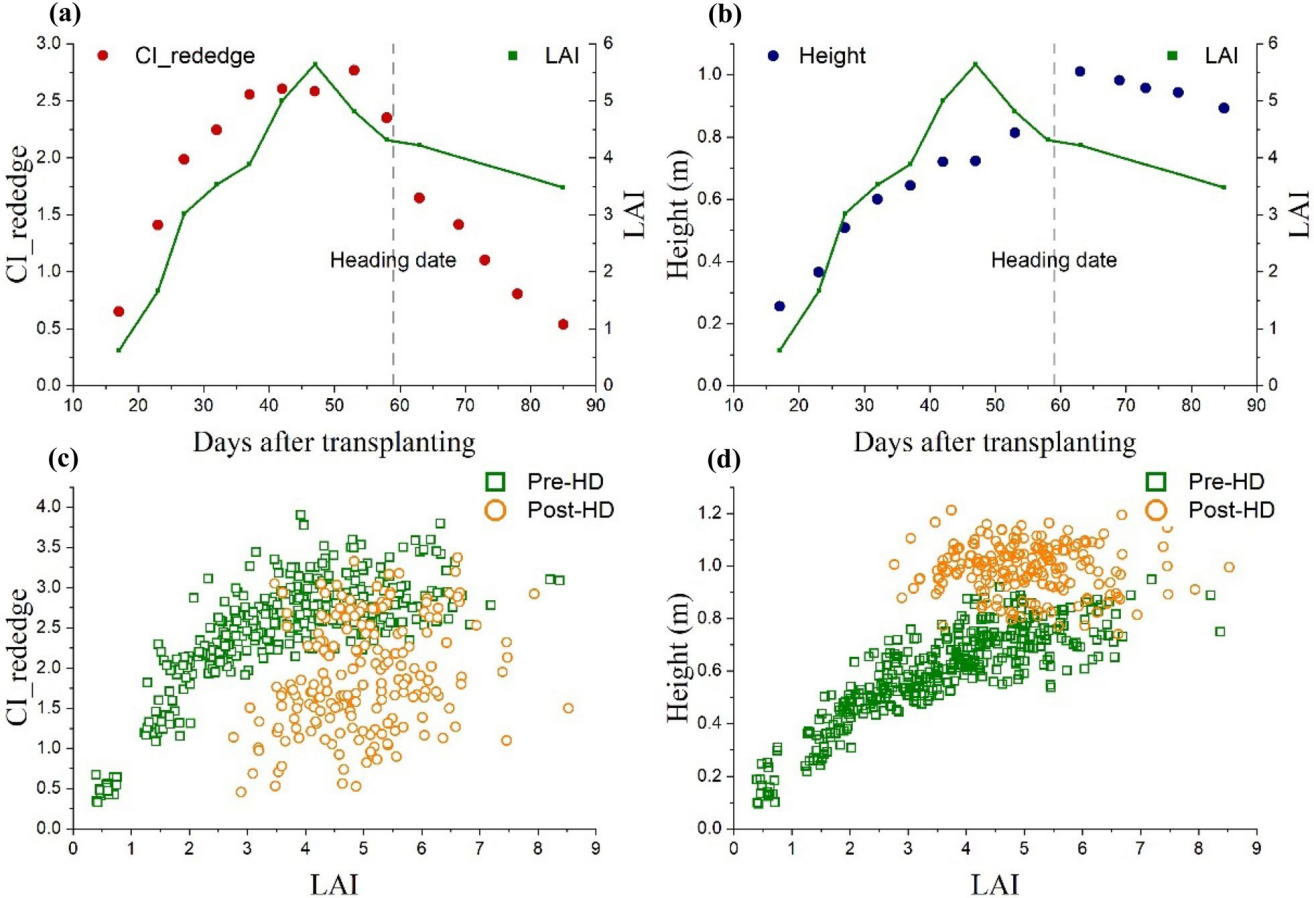
Temporal behaviors of measured LAI vs. **a** CI_red edge_ and **b** canopy height, and the LAI variation plotted with **c** CI_red edge_ and **d** canopy height during the rice entire growing season

The temporal behaviors of rice LAI, VI (e.g., CI_red edge_), and canopy height were compared throughout the entire growing season (Fig. 5a, b). From the beginning of the season, rice LAI sharply increased to peak value around 10 days before heading date and then gradually decreased afterwards. While CI_red edge_ increased to a relatively high value approximately 20 days before heading date. It remained invariant or slightly increased until the heading date and then sharply decreased towards the end of the season. In contrast to CI_red edge_, rice height continued to increase approximately 5 days after the heading date and then it decreased a little. The different temporal behaviors of rice LAI, height, and VI may result in hysteresis effects existed on the relationship LAI vs. VI as well as the relationship LAI vs. height (Fig. 5c, d). Noted that the hysteresis on LAI vs. VI relationship and LAI vs. height relationship was opposite in direction, i.e., for the same rice LAI the canopy height was higher but VI was lower during the post-heading stages than those during the pre-heading stages (Fig. 5c, d). This may offer an opportunity to improve LAI estimation model based on both VI and canopy height information to minimize hysteresis between pre-heading and post-heading stages.

### LAI estimation based on remotely sensed VI and canopy height

**Fig. 6:**
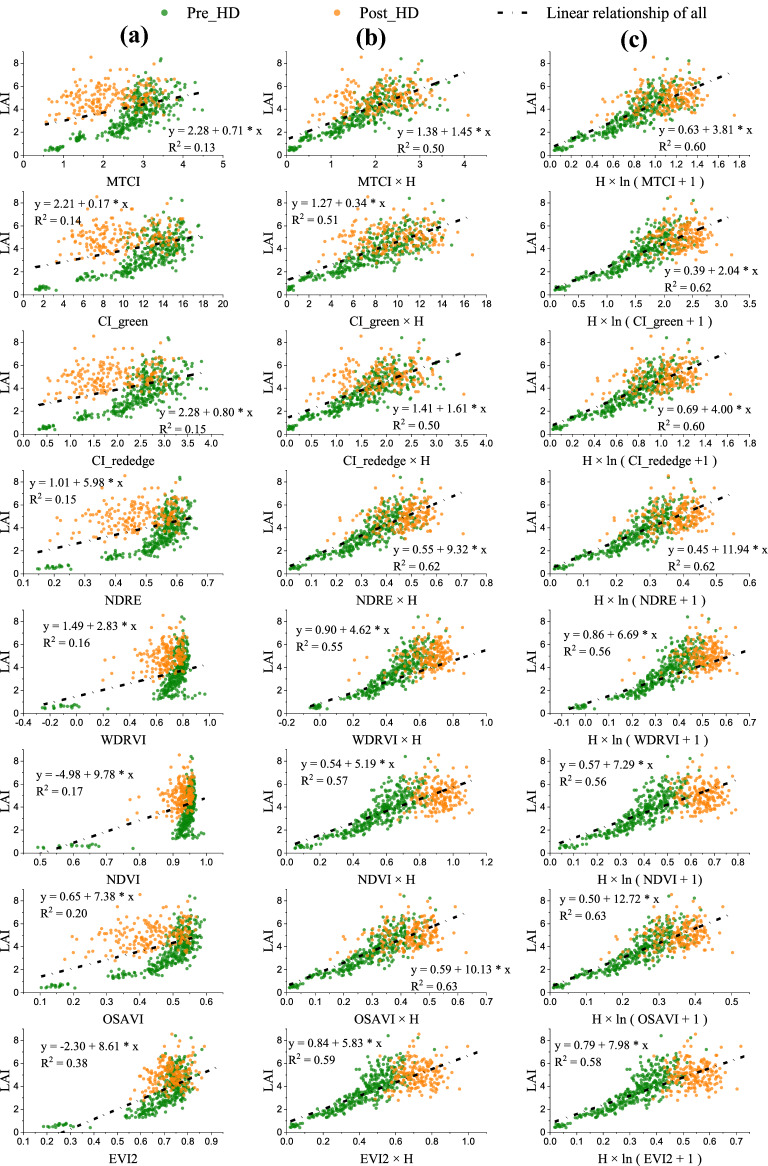
Three models, **a** LAI vs. VI, **b** LAI vs. H × VI and **c** LAI vs. H × ln(VI + 1), were tested for rice LAI estimation throughout the entire growing season for eight tested indices

Three models, LAI vs. VI, LAI vs. H × VI and LAI vs. H × ln(VI + 1), were tested for rice LAI estimation throughout the entire growing season. It is found that the hysteresis between pre-heading and post-heading stages was considerably diminished on LAI vs. H × VI and LAI vs. H × ln(VI + 1) models (Fig. 6). Moreover, the indices saturated to moderate-to-high LAI (e.g., NDVI, NDRE and OSAVI) became much more sensitive to the wide range of LAI variation for H × VI and H × ln(VI + 1) model.

The k-fold cross validation procedures were applied to develop algorithms estimating rice LAI throughout the entire growing season based on VI, H × VI and H × ln(VI + 1) model. For all tested indices, the model using both H and VI information was much more accurate than the model using solely VI. When canopy height included in the model, the estimation error significantly decreased by more than 20% (Table 4). Generally, the model based on H × ln(VI + 1) worked a little better than the model based on H × VI. For tested indices H × VI model can give LAI estimations with RMSE below 1.1 and CV below 27%, and H × ln(VI + 1) model can give LAI estimations with RMSE below 1.02 and CV below 25%.

**Table 4:** Root mean square errors (RMSE) and coefficient of variation (CV) of LAI estimation based on VI, H × VI and H × ln(VI + 1) model using ten-fold cross-validation

	VI		VI × H		H × ln(VI + 1)	
	RMSE	CV (%)	RMSE	CV (%)	RMSE	CV (%)
MTCI	1.44	35.1	1.09	26.6	0.97	23.8
${\text{CI}}_{{{\text{green}}}}$	1.43	34.9	1.08	26.3	0.95	23.1
${\text{CI}}_{{{\text{red edge}}}}$	1.43	34.9	1.09	26.6	0.98	23.8
NDRE	1.42	34.7	0.96	23.4	0.95	23.2
WDRVI	1.42	34.6	0.95	23.1	0.95	23.3
NDVI	1.41	34.4	1.01	24.7	1.02	24.9
OSAVI	1.38	33.7	0.94	23.0	0.94	22.9
EVI2	1.20	29.4	0.98	23.9	0.99	24.1

**Fig. 7:**
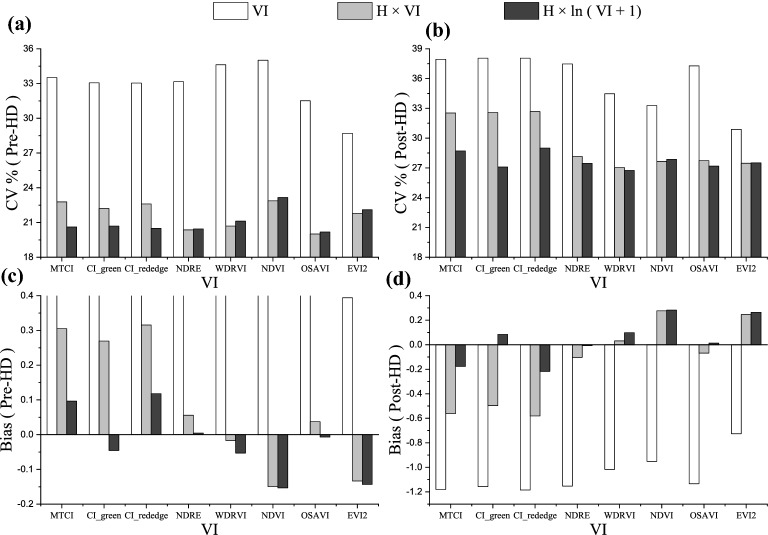
Using one algorithm during entire rice growing seasons for LAI estimation based on VI, H** × **VI and H** × **ln (VI + 1) models with **a** coefficient of variation (CV) for pre-heading stages, **b** CV for post-heading stages, **c** Bias for pre-heading stages and **d** Bias for post-heading stages

Using one algorithm to estimate rice LAI throughout the entire growing season, the estimation errors of pre-heading and post-heading stages were compared for three models. For both phenology stages, the inclusion of canopy height can obviously improve the model accuracy with CV decreased by 6.60–13.50% for pre-heading stages and by 3.37–10.96% for post-heading stages (Fig. 7a, b). When developing the algorithm using solely VI, for all indices LAI would be over-estimated at pre-heading stages and under-estimated at post-heading stages (Fig. 7c, d). While the use of H × VI and H × ln(VI + 1) model could greatly reduce the estimation bias for both phenology stages. Using VI model, the average bias of over-estimation was 0.39–0.64 for pre-heading stages and the average bias of under-estimation was 0.72–1.18 for post-heading stages. Using H × VI and H × ln(VI + 1) model, no obvious over- or under-estimation effects were consistently observed, and the average bias were considerably reduced for both phenology stages with $\left| {{\text{Bias}}} \right|$ < 0.32 for pre-heading stages and $\left| {{\text{Bias}}} \right|$ < 0.58 for post-heading stages (Fig. 7c, d). Combining canopy height information, NDRE, WDRVI and OSAVI could estimate LAI with bias under ± 0.1 during the entire growing season.

**Fig. 8:**
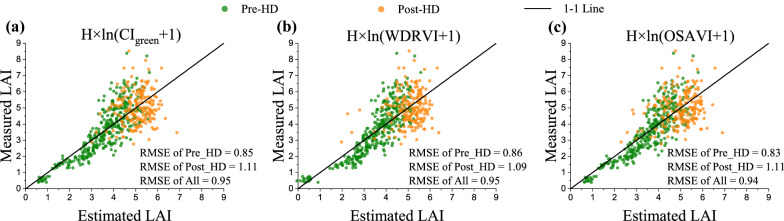
Comparison of estimated and measured LAI based on H** × **ln(VI + 1) model using **a** CI_green_, **b** WDRVI and **c** OSAVI

Generally, the model based on H × ln(VI + 1) worked a little better than the model based on H × VI for most indices. CI_green_, WDRVI and OSAVI were the best estimating LAI in various rice cultivars throughout the entire growing season with green, red and red edge bands, respectively (Fig. 8):

$${\text{LAI}} = 2.04 \times {\text{H}} \times \ln ({\text{CI}}_{{{\text{green}}}} + 1) + 0.40,\,\,{\text{RMSE}} = 0.95\;{\text{CV}} = 23.1\%$$

$${\text{LAI}} = 9.02 \times {\text{H}} \times \ln (WDRVI + 1) + 0.55,\,{\text{RMSE}} = 0.95\;{\text{CV}} = 23.3\%$$

$${\text{LAI}} = 12.72 \times {\text{H}} \times \ln ({\text{OSAVI}} + 1) + 0.50,\,{\text{RMSE}} = 0.94\;{\text{CV}} = 22.9\%$$

## Discussions

The indices tested in this study have been widely applied for estimating vegetation biophysical parameters (e.g., LAI, canopy Chl, biomass) in many crop species [14, 25, 37]. Hysteresis was also reported in canopy Chl vs. VI relationship between vegetative and reproductive stages in maize and soybean, but such hysteresis was not significant and using one relationship for the entire growing season could estimate canopy Chl with acceptable accuracy [37]. However, our study found that LAI vs. VI relationship in rice for the entire growing season had high uncertainties (Table 3, Fig. 3). Rice has its distinct canopy structure during the growing season [43]: After transplanting the seedling, plant height gradually increases and more leaves develop at regular intervals. The tillering stage extends from the appearance of the first tiller until the maximum number of tillers. At this period, the plant stem lengthens but stops growing just before panicle initiation. As a bulging of the leaf stem conceals the developing panicle, called the booting stage, the rice plant is entering its reproductive phase from vegetative phase. The tip of the developing panicle then emerges from the stem and continues to grow, and the heading stage is coming when the panicle is fully visible on top canopy. When rice enters ripen phase, plant growth can be subdivided into milky, dough and maturity stages based on the texture and color of growing grains. At milky stage, grain is milky and reaches final size, but the panicles are still green; at dough stage, grain is gradually dried and solid and panicles turn to yellow; At maturity stage, grains are very hard and waited to be harvested [79]. The pre-heading stages defined in this study includes transplanting, tillering, booting stages while the post-heading stages includes heading, milky, dough and maturity stages.

**Fig. 9:**
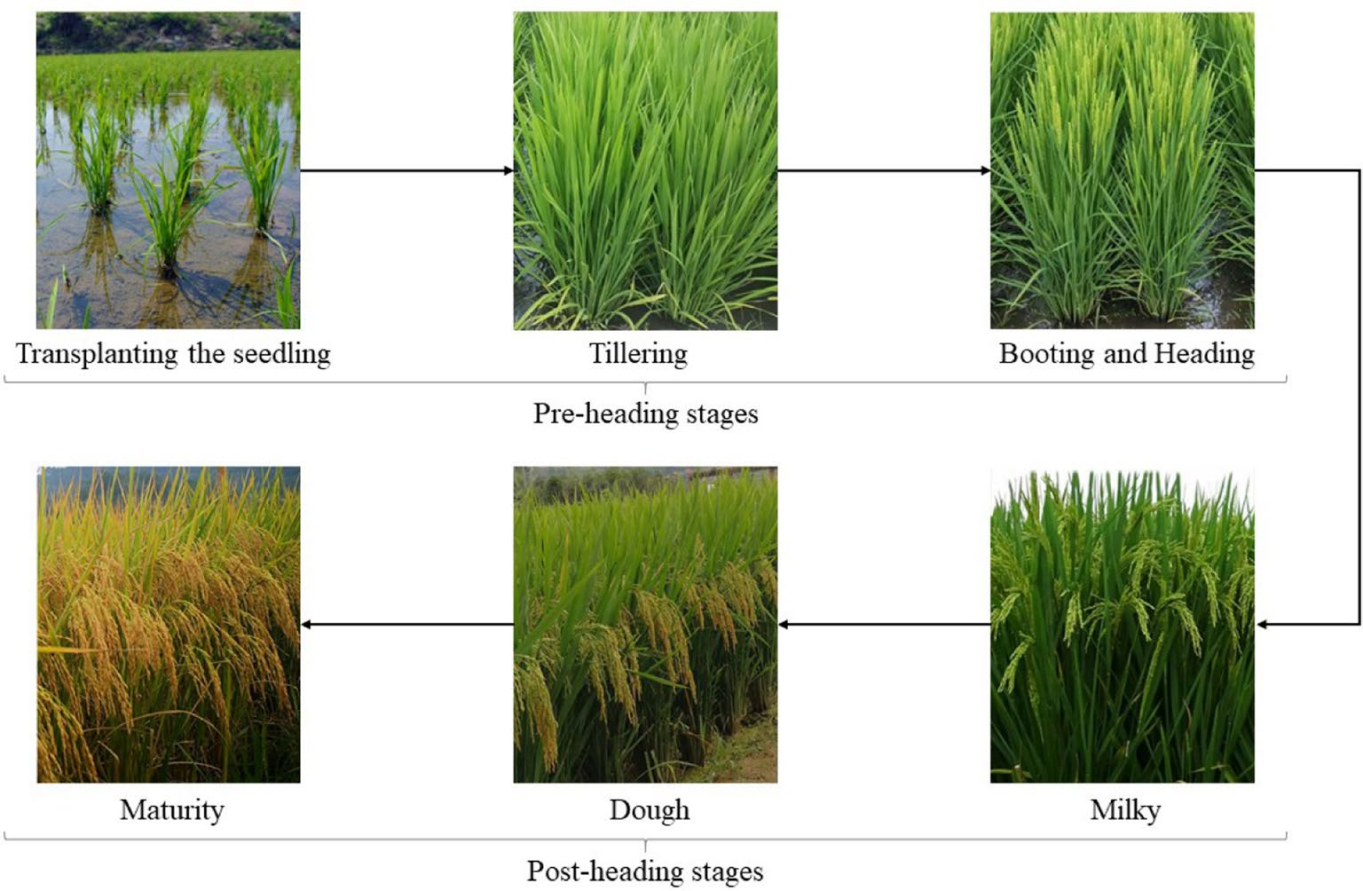
Phenology stages of rice growth cycle

Canopy reflectance in the visible region is determined by pigment absorption, and reflectance in NIR region is mainly affected by canopy structure [80, 81]. Photosynthesis mainly takes place inside the leaf, so visible radiation absorbed by leaf is a lot higher than that by panicle. Panicles are apparent on top canopy since booting and heading stages and they turn into yellow during rice post-heading stages. As rice ripening, panicles droop due to increased grain weight and may partially cover the leaves below (Fig. 9). The dense and droopy panicles can prevent the light penetration inside the canopy thus causing the rise of visible reflectance. Therefore, for the same LAI value visible reflectance in the period before rice heading was much lower than after heading when panicles occupying more than half of top canopy (Fig. 4). In addition, it is reported with the same LAI, the dark green leaves in the vegetative stage have much higher chlorophyll content than in the reproductive stage with leaf senescence [82], so leaves in pre-heading stages can absorb more visible light than in post-heading stages thus resulting in lower reflectance.

In NIR region, canopy reflectance tended to decrease during the post-heading stages as plant becoming senescent. When rice entering ripening phase, panicles tend to droop with the maturity of growing grain (Fig. 9), which greatly changes the canopy architecture making NIR reflectance fluctuating with high uncertainties (Fig. 4).

With the same LAI, the visible reflectance of rice canopy was higher at post-heading stages than pre-heading stages, while NIR reflectance of rice canopy was similar or lower at post-heading stages. Since our tested VIs were calculated based on the ratio or difference between NIR and visible reflectance, they behaved much lower at post-heading stages than at pre-heading stages causing hysteresis of VI vs. LAI relationship between two stages (Fig. 3). The hysteresis for normalized difference indices was smaller than that for ratio indices (Fig. 3), and hysteresis for EVI2 and OSAVI appeared relatively smallest (Fig. 3g, h) since the constant used in such VI formula may somewhat attenuate the reflectance difference at different phenology stages.

As rice grows before heading, remotely sensed VI closely followed LAI variation (Fig. 3) and the relationships VI vs. LAI are in accordance with the observations in many previous studies. The indices derived from the ratio of NIR and red reflectance (e.g., NDVI, EVI2 and WDRVI) were saturated to moderate-to-high LAI since red reflectance behaved almost invariant for vegetation with moderate-to-high density [19, 28]. The normalized indices (e.g., NDVI, NDRE, OSAVI) were insensitive to high LAI variation due to much higher NIR reflectance than visible reflectance for vegetation having high chlorophyll content [83]. The ratio indices using red edge or green reflectance seemed more linearly related to LAI since green and red edge reflectance is more sensitive to the wide range of LAI variation [84]. With the emergence of panicle, however, samples obviously deviated from the relationship which the pre-heading samples followed. With the same LAI, VI of pre-heading stages can be twice higher than that of post-heading stages. Using the algorithm developed by samples collected before heading, LAI at post-heading stages would be significantly underestimated. That’s why the R^2^ of VI vs. LAI relationship before rice heading is high (above 0.8) but it dramatically decreased as more and more post-heading samples included.

However, it is unrealistic to firstly separate rice into pre-heading and post-heading samples and then develop the relationships respectively. Extensive field work has to be conducted to record the heading date mainly based on visual inspection, which can be somewhat subjective. The method to estimate rice LAI for the entire growing season, not requiring pre-knowledge of heading date and algorithm re-parameterization for different phenology stages, is imperative to be developed especially for rice breeding experiments with various hybrids having different heading dates. In addition, for images of which the pixel size larger than a single cultivar plot, the model applicable for the entire growing season and for different rice cultivars is essential to estimate rice LAI efficiently and accurately.

This study explored to use rice canopy height improving VI model to minimize hysteresis of LAI estimation algorithms between two different phenology stages. Canopy height can be accurately and remotely retrieved from stereo-observation images [85, 86] and it has been used to estimate vegetation growth parameters (e.g., LAI, biomass) in some research [60, 63, 86, 87]. In vegetative stage as plant grew canopy height as well as VI increased, so they both positively correlated with rice LAI (Fig. 5). In this period, more number of developed leaves and the increase in leaf chlorophyll content contributed to maximize sunlight absorption. So VI sharply increased and then maintained at high level from late tillering stage to early heading stage. Around booting stage before rice heading, LAI reached almost maximum as flag leaf stretching completely. During heading stage, the elongated top internodes, together with the panicle exsertion, made the canopy height continue to increase late after the heading date. After heading stage as more and more green leaves turning to yellow and becoming senescent, VI sharply decreased due to the decline of leaf chlorophyll content and light absorption; LAI gradually decreased due to the shrinking of aging leaves; canopy height began to decrease slightly since milky stage because the increase in grain size and weight made more panicles droopy.

In this case, canopy height may be indicative to panicle development, which can be used as an addition to VI for rice LAI estimation. For the same LAI, canopy height was higher in the period of post-heading than pre-heading (Fig. 5d), while VI was lower at post-heading stages (Fig. 5c). Thus solely using VI for LAI estimation of the entire season, LAI could be over-estimated at pre-heading stages while under-estimated at post-heading stages (bias > 0 for pre-heading stages and bias < 0 for post-heading stages—Fig. 7). The production of VI and canopy height may compensate the hysteresis of VI vs. LAI relationship between pre- and post-heading stages. Moreover, during post-heading stages, the rate of VI change was much greater than LAI change while canopy height slightly decreased. So canopy height could somewhat adjust VI to follow LAI variation more closely. For all tested indices, the use of canopy height in VI model can significantly increase estimation accuracy (Table 4) and no obvious hysteresis was observed between two stages (Fig. 6). Moreover, the indices which appeared nonlinearly related to LAI with pre-heading samples (e.g., NDVI, OSAVI) became much more linearly related to LAI (Fig. 6) in H × VI and H × ln(VI + 1) model. By including canopy height in model, LAI estimation accuracy was significantly increased for both stages with CV and bias greatly reduced at pre and post-heading stages (Fig. 7).

This study clearly shows that crop phenology may significantly affect canopy spectra and structure during its reproductive stage, especially for the crop with conspicuous flowers, fruits or grains having very different spectral or structural features from leaves. So phenology factor need to be considered in reflectance-based models for estimating crop biophysical parameters throughout its growing season. The method developed in our study is very simple for rice LAI estimation by including canopy structure information as a good addition to spectral information, which can be effectively applied in remotely sensed images with two or three traditional visible and NIR bands. By this method, it is not necessary to create new or complicated indices particularly for considering panicle factor but just incorporate rice height information into existing widely used VI models. The algorithms used in this study are linear regressions that worked efficiently, so we don’t need to go for sophisticated algorithms (e.g., machine learning method) with big computation and requiring hyperspectral data which is sometimes costly or unavailable. Since VI and canopy height can be obtained by various UAV platforms at low cost, our method can be used for routine monitoring of rice LAI during the entire growing season. This can provide a rapid and quantitative way to evaluate rice growth at large scale, especially beneficial for high-through screening and selecting target crop in rice breeding studies having a large number of cultivars under different field conditions. But we realize that this study was only tested in 48 rice cultivars, and our model will be tried in much more rice cultivars all around the world. Also, our method was developed in rice with UAV images, the future work includes applying and improving it for other crop species with potential high-resolution satellite data.

## Conclusions

In this study, we developed a method to remotely estimate LAI using UAV-retrieved canopy reflectance and height for different rice cultivars during the entire growing season. Several widely used VIs were calculated from canopy reflectance, and significant hysteresis was observed in VI vs. LAI relationship between rice pre-heading and post-heading phenology stages. For the same LAI, canopy VI was higher while canopy height was lower at post-heading stage than at pre-heading stage. The model based on the product of canopy reflectance and height effectively reduced hysteresis effect due to phenology difference and obviously improved the accuracy of rice LAI estimation throughout the entire growing season. The model using one algorithm during the whole growing season with OSAVI and canopy height can estimate rice LAI with RMSE under 1.1 for both pre- and post-heading stages, not requiring algorithm re-parameterization for different phenology stages.

## Data Availability

The datasets used and analyzed during the current study may be available upon the agreement from the corresponding author on reasonable request.
